# Satellite-Dominated Sulfur L_2,3_ X-ray Emission of Alkaline Earth Metal Sulfides

**DOI:** 10.1021/acsomega.2c07228

**Published:** 2023-01-23

**Authors:** Lothar Weinhardt, Dirk Hauschild, Oliver Fuchs, Ralph Steininger, Nan Jiang, Monika Blum, Jonathan D. Denlinger, Wanli Yang, Eberhard Umbach, Clemens Heske

**Affiliations:** † Institute for Photon Science and Synchrotron Radiation (IPS), Karlsruhe Institute of Technology (KIT), Hermann-v.-Helmholtz-Platz 1, Eggenstein-Leopoldshafen 76344, Germany; ‡ Institute for Chemical Technology and Polymer Chemistry (ITCP), Karlsruhe Institute of Technology (KIT), Engesserstraße 18/20, Karlsruhe 76128, Germany; § Department of Chemistry and Biochemistry, University of Nevada, Las Vegas (UNLV), 4505 Maryland Parkway, Las Vegas, Nevada 89154, United States; ∥ Experimentelle Physik VII, Universität Würzburg, Am Hubland, Würzburg 97074, Germany; ⊥ Advanced Light Source (ALS), Lawrence Berkeley National Laboratory, 1 Cyclotron Road, Berkeley, California 94720, United States; # Chemical Sciences Division, Lawrence Berkeley National Laboratory, 1 Cyclotron Road, Berkeley, California 94720, United States

## Abstract

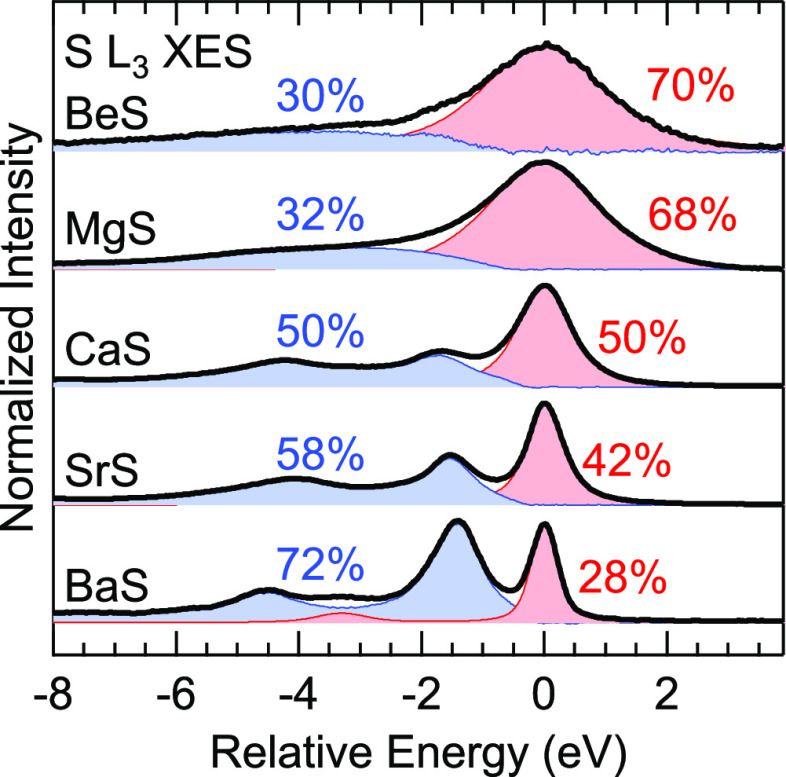

The sulfur L_2,3_ X-ray emission spectra of the alkaline earth metal sulfides BeS, MgS, CaS, SrS, and BaS are investigated and compared with spectra calculations based on density functional theory. Very distinct spectral shapes are found for the different compounds. With decreasing electronegativity of the cation, that is, increasing ionic bonding character, the upper valence band width and its relative spectral intensity decrease. These general trends are qualitatively reproduced by the spectra calculations, which give quite an accurate description of the spectral shapes in the upper valence band region. On the low energy side of the sulfur 3s → 2p transition dominating the spectra, we find strong satellites caused by “semi-Auger” decays involving configuration interaction. These satellites, previously believed to be energetically forbidden for sulfur L_2,3_ emission and only observed for the L_2,3_ emission of Cl to Cr, increase in intensity as the bonding character becomes more ionic and dominate the spectra for SrS and BaS. The intensities, energies, and widths of the satellites vary strongly between the investigated compounds, giving a very specific spectral fingerprint that can be used for speciation analysis.

## Introduction

Soft X-ray emission spectroscopy (XES) is a powerful technique to study the electronic and chemical structure of solid, liquid, and gaseous materials. In many cases, the spectra are dominated by valence-to-core emission with a single valence hole final state, thus probing the local partial density of valence band states.^1^ Here, “local” reflects the necessity of a wave function overlap between the involved core and valence levels, while “partial” refers to the (dipole) selection rules for angular momenta. Briefly, XES thus probes the valence band structure (which strongly reflects chemical bonding) from the viewpoint of a selected core level/element. This selectivity makes it very useful and sensitive for chemical speciation with unique “fingerprints” of different compounds.^2−5^

Due to the finite duration of the X-ray emission process, the dynamic reaction of the system can lead to a multitude of additional features.^6^ This is most prominent (and can even be somewhat controlled) with resonant excitation (resonant inelastic X-ray scattering), but also occurs for non-resonant excitation. Processes include multiply excited states^7−9^ and lifetime vibrational interference^10,11^ in molecules, which can lead to dissociated final states.^12,13^ Most relevant for the present study, satellite lines can also appear caused by “semi-Auger” decays involving configuration interaction and leading to double valence hole final states.^14−19^ These satellites have been observed for 3s → 2p transitions in the emission spectra of Cl to Cr, with intensities of up to approx. 30% of the total intensity.^19^ For S, these transitions are believed to be energetically forbidden.^19^

In this paper, we demonstrate that these satellites are also present in the S L_2,3_ emission of alkaline earth metal sulfides, can amount up to 70% of the total intensity, and vary as a function of the specific compound. The latter results in very specific fingerprints, which allows a highly sensitive speciation of the different compounds. This is of particular interest in device applications, where alkaline earth metal sulfides play important roles, for example, for batteries^20−22^ or as materials for photodiodes in the ultraviolet range.^23,24^ Hence, enabling deeper insights into their electronic structure by enabling a correct and detailed interpretation of XES spectra from, for example, buried layers and interfaces is also of significant interest for device applications.

## Experimental and Computational Methods

Sulfide powders were purchased from Alfa Aesar (BeS, 99% purity; SrS, 99.9%; BaS, 99.7%), Beantown Chemicals (CaS, 99%), and ALB Materials Inc. (MgS, 99.9%). BeS, CaS, SrS, and BaS powders were pressed on UHV compatible conductive carbon tape (SPI Supplies) and mounted on a sample holder in an N_2_-filled glove box (CaS, SrS, and BaS) or in air (BeS), respectively. The CaS, SrS, and BaS samples were transported in a N_2_-filled container to the beamline and introduced into the experimental chamber without air exposure (using an N_2_-filled glove bag). For the MgS sample, the powder material was pressed to a thin pellet in an Ar-filled glove box. Subsequently, it was transported to the beamline in an Ar-filled sample container that was directly connected to the experimental station and allowed for a sample transfer without any air exposure.

Experiments were performed in three different setups. The BeS data were collected in the former soft X-ray fluorescence endstation with a high-resolution Rowland-circle spectrograph,^25^ installed at Beamline 8.0 of the Advanced Light Source (ALS), Lawrence Berkeley National Laboratory (Berkeley, USA). The CaS, SrS, and BaS data were collected in the Solid And Liquid Spectroscopic Analysis (SALSA) roll-up experimental station^26^ at Beamline 8.0.1 of the ALS, using SALSA’s high-transmission spectrometer.^27^ The MgS data were measured at the X-SPEC beamline^28^ at the KIT Light Source, using a next-generation high-transmission soft X-ray spectrometer.^29^ All data were collected with a spectrometer resolution of better than 0.2 eV. For the CaS, SrS, and BaS samples, the energy scales of beamline and spectrometer were carefully calibrated as described in ref (5), which gives an emission energy scale with an absolute uncertainty of 0.1 eV and a relative uncertainty of 0.03 eV between the measurements. The energy scale of the BeS spectra was adjusted to that of the CaS, SrS, and BaS data by CdS reference measurements, giving a relative uncertainty of 0.2 eV. The energy scale of the MgS spectra was adjusted to that of the CaS, SrS, and BaS data by CaSO_4_ reference measurements, giving a relative uncertainty of 0.1 eV. The CaS, SrS, and BaS spectra also contain contributions from C K and O K emission from the carbon tape substrate excited by higher order light of the beamline and collected in higher orders of the spectrometer. These contributions were carefully removed, as described in the supporting information.

Band structures, projected density of states (PDOS), and XES spectra were calculated with WIEN2k, which is based on the full-potential augmented plane wave plus local orbitals (APW + lo) method to solve the Kohn-Sham density functional theory (DFT) equations.^30^ XES spectra were calculated in the generalized gradient approximation (GGA),^31^ the dipole approximation, and Fermi’s Golden Rule in the formalism described by Schwarz et al.^32−34^ 1,000 and 10,000 *k* points were used for the PDOS and the XES spectra, respectively. The crystal structures were obtained from the Materials Project Database.^35^ A more detailed description of the approach can be found in ref (5).

## Results and Discussion

**Figure 1:**
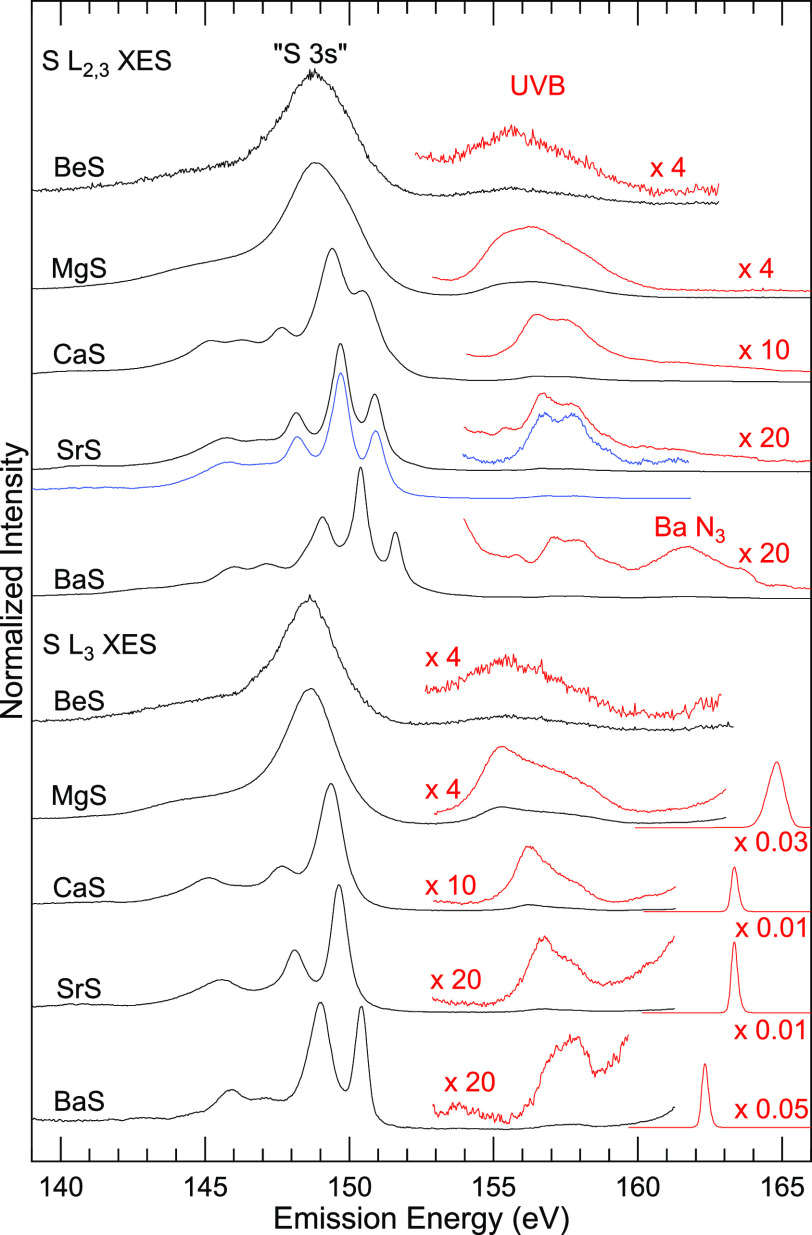
S L_2,3_ (top) and S L_3_ (bottom) XES spectra of BeS, MgS, CaS, SrS, and BaS. The S L_2,3_ spectra were excited with 180 eV (BeS), 185.2 eV (MgS), and 184.6 eV (CaS, SrS, and BaS). The S L_3_ spectra were excited with 165 eV (BeS), 164.8 eV (MgS), 163.3 eV (CaS and SrS), and 162.3 eV (BaS). Spectral regions shown in red (upper valence band, UVB, and Rayleigh line) were multiplied by the given factors. The blue spectrum is a 1:2-weighted sum of two L_3_ XES spectra of SrS, shifted relative to each other by 1.20 eV.

The S L_2,3_ and S L_3_ XES spectra of the investigated alkaline earth metal sulfides are presented in Figure 1. While the S L_2,3_ XES spectra (top half of Figure 1) are excited well above the absorption edges, the S L_3_ XES spectra (bottom half) are excited between the S L_2_ and L_3_ absorption edges. The upper valence band (UVB) region as well as the elastic lines are shown in red (with the given magnification factors).

As the blue spectrum in Figure 1 shows for the example of SrS, the S L_2,3_ XES spectra can generally be described as a weighted sum of two spectra. To create the weighted sum, the elastic line is subtracted from the S L_3_ spectrum, two copies are created and weighted with an L_2_:L_3_ intensity ratio of 1:2, and the L_2_ spectrum is shifted to higher emission energies by the S 2p spin-orbit splitting of 1.20 eV.^36,37^ The good agreement indicates that resonant effects are comparably weak in the present case; in the following, we will thus treat the S L_3_ spectra as “non-resonant” spectra (but with one of the spin-orbit split core holes “turned off”). For the S L_2,3_ spectrum of BaS, the excitation energy is sufficient to also excite the N_3_ edge of Ba—a corresponding feature is found at an emission energy of ∼162 eV. In contrast, the S L_3_ spectrum of BaS is excited below the Ba N_3_ edge.

Two main spectral regions can be distinguished: below an emission energy of 153 eV, we find transitions involving the S 3s-derived states (denoted “S 3s”), while transitions above 153 eV involve S 3p-derived states (in the UVB region). As expected, the intensity of the latter is very weak, owing to the dipole selection rules for p core holes and the predominantly p-type symmetry of the involved valence state wave functions. For an isolated S^2–^ ion, the intensity in this region would fully vanish; the remaining intensity is thus indicative of the partially covalent bonding character of the compounds, which will be discussed in more detail below. The “S 3s” region varies strongly between the different compounds. While the S L_3_ XES spectra of BeS and MgS show only one broad structure with low energy tails in this region, the other three sulfides show three to four well-separated peaks. The relative intensities of these peaks are very different for the different compounds. Furthermore, the “S 3s” emission shifts to higher energies and the width of the highest-energy peak decreases with increasing atomic number of the cation (top to bottom).

**Figure 2:**
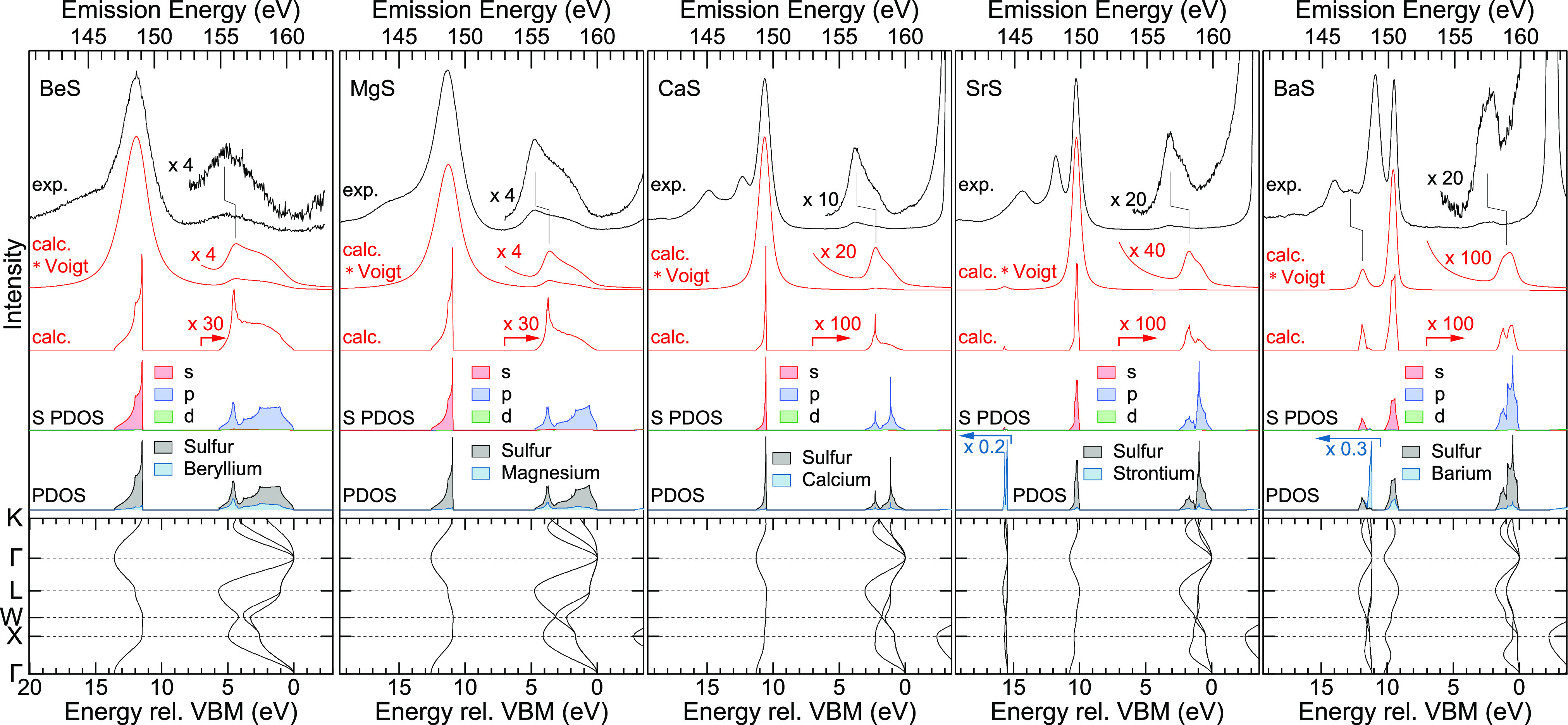
Analysis of the S L_3_ spectra of (from left to right) BeS, MgS, CaS, SrS, and BaS. From bottom to top: calculated *k*-resolved band structures, density of states projected onto the different atomic species (PDOS), density of states projected onto s, p, and d symmetry for the S atom (S PDOS), calculated S L_3_ XES spectra (“calc.,” red), calculated spectra convoluted with a Voigt profile to account for experimental and lifetime broadening (“calc. × Voigt,” red), and experimental spectra (top, black). For better visibility, the S PDOS and the calculated L_3_ spectra are shown magnified by the given factors for energies smaller than 7 eV [relative to the valence band maximum (VBM)]. Likewise, the Sr and Ba PDOS were multiplied by the given factors for energies larger than 15 and 10.5 eV, respectively. The energy scales of the calculation and the experiment were arbitrarily aligned with respect to the peak with highest emission energy in the “S 3s” region.

Next, we compare the S L_3_ spectra with DFT electronic structure and spectra calculations in Figure 2. At the bottom, the calculated band structure of the five compounds is shown with the energy relative to the VBM on the abscissa and the reciprocal (*k*-) space on the ordinate. Qualitatively, the *k*-dependence of the bands is very similar for all compounds and determined by their rocksalt structure and ionic nature (see the derivation of universal valence bands for rocksalt-type compounds in ref (38)). Quantitatively, the widths of the bands vary strongly between the different compounds, which will be discussed below. Furthermore, additional bands appear for SrS (at ∼15.5 eV rel. VBM) and BaS (at ∼11.5 eV rel. to VBM), which are derived from the Sr 4p and Ba 5p atomic states, respectively. In the case of BaS, the additional band also hybridizes with the S 3s-derived band, changing its *k*-dependence and giving rise to a small peak in the experimental spectra of BaS in Figure 1 (at 147.1 eV).

Above each band structure, the density of states projected onto the respective atom types (PDOS) is shown. As expected by the large difference in electronegativity, the PDOS are dominated by S, while only a small PDOS is observed at the cations. The exceptions are the Sr 4p- and Ba 5p-derived bands, which are mainly localized at the respective cation. Above the PDOS, the PDOS of sulfur (S PDOS) is depicted, now also projected to the orbital angular momentum (s, p, and d). We find that the S 3s-derived bands have nearly pure s symmetry (red), while the UVBs exhibit nearly pure p symmetry (blue) with a minor admixture of s (red) and d (green) symmetry. With this, the calculated S L_3_ XES spectra plotted above (“calc.”) are dominated by emission from the S 3s-derived bands, and only low intensity is found in the UVB regions. For a direct comparison with the experimental data shown in black at the top of the graphs, we have convoluted the calculated spectra with Voigt functions to account for experimental and lifetime broadening (“calc. × Voigt”). The widths were adjusted to fit the experimental spectra.

**Figure 3:**
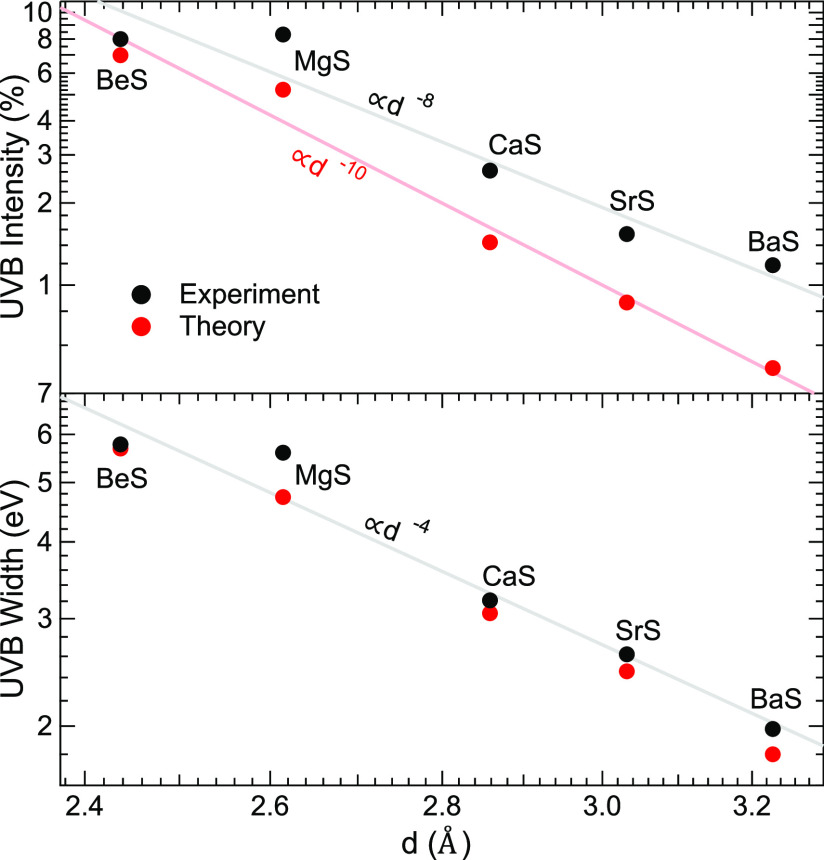
Experimental (black) and calculated (red) width (lower panel) and relative intensity (upper panel) of the upper valence band (UVB) as a function of the lattice constant *d* of the investigated sulfides. The axes are shown on logarithmic scales, and the gray and red lines are fits of the data with power functions (integer exponents are given), which serve as guides to the eye.

A good agreement between the line shapes of the calculated and experimental spectra is achieved in the UVB regions. This is further analyzed by plotting the widths and relative intensities of the UVBs in Figure 3. The experimental widths were determined by stretching the UVB of the calculated spectra (“calc. × Voigt”) to best agree with the experimental ones and then multiplying the calculated band widths with the respective factors. We find that, with the exception of MgS, the calculation only slightly underestimates the experimental band widths (i.e., the factors are only slightly larger than unity, ranging from 1.02 for BeS to 1.18 for MgS) and nicely reproduces the observed significant decrease of the band widths with increasing lattice constant. This decrease is caused by the decreasing electronegativity (increasing ionic radius) of the cation with increasing period in the periodic table, making the bonding character in the here-studied sulfides increasingly ionic. With increasing ionic radius of the cation, the lattice constant of the sulfides also increases, making the latter a reasonable parameter in Figure 3. In fact, a simple analytical relationship between the band width and the lattice constant *d* for rocksalt-type compounds by Pantelides predicts a *d*^–2^ dependency.^38^ Our calculations and experimental data better fit to a *d*^–4^ dependence, as indicated by the gray line in the lower panel of Figure 3.

In addition to the reduction in band width, we observe a strong decrease of the relative intensity of the UVB with increasing lattice constant (again, for MgS, the decrease is smaller than predicted by the overall trend). In this case, however, we find a significant discrepancy between our calculation and the experimental data. The experimental UVB intensity is much higher and decreases much less rapidly (approximately proportional to *d*^–8^) than predicted by the calculation (∝*d*^–10^), which can be explained as follows. From the calculated PDOS, we find that the decrease of the UVB intensity in the S L_2,3_ emission is caused by the UVB becoming more p-like (and thus the corresponding transitions becoming increasingly dipole-forbidden) when the compounds become more ionic. This, in turn, suggests that some degree of symmetry breaking occurs in the experiment, on the time scale of the X-ray emission process. This “reaction” of the electronic valence structure to the presence of the localized core hole is an effect that is not included in the calculation. Since the UVB is highly dominated by states of p symmetry, such an effect does not need to be very strong to cause the observed significant impact on the S L_2,3_ emission intensity.

In the “S 3s” spectral region, the calculation predicts only one peak for BeS, MgS, CaS, and SrS and one main peak and a weak second line for BaS. In contrast, the experimental spectra show a much richer structure. These additional features are satellite lines, attributed to final states not accounted for in our calculation. The calculation purely accounts for single valence hole final states, that is, the “S 3s”^–1^ final state in this spectral region. The calculated energy differences between the UVBs and the 3s-derived bands of CaS, SrS, and BaS suggest that the middle peak (i.e., at 147.6 eV for CaS, 148.1 eV for SrS, and 149.0 eV for BaS) could correspond to the “3s”^–1^ final state; a satellite would then be present at higher emission energy. Indeed, such high-energy satellites have been observed in small molecules and are caused by multiple excitations.^8,9^ However, in our case, this type of satellite can be ruled out, since all peaks are present for L_3_-only excitation, where the necessary excess energy for double excitation is not available. Furthermore, GW calculations of CaS^39^ indicate that the binding energy of the S 3s-derived band might be overestimated when using the local density approximation or, like in our case, the GGA. Lastly, electron correlation effects in the final state might also lead to a shift in energy. Taking this into account, we attribute the “S 3s”^–1^ final state to the peaks with the highest emission energy in the “S 3s” region of the spectra. In Figure 2, the energy scales of the calculated and experimental spectra were aligned accordingly. With this, the additional feature at ∼11.5 eV rel. to VBM (147.1 eV in Figure 1) in the spectrum of BaS can be assigned to emission involving the Ba 5p-derived bands.

**Figure 4:**
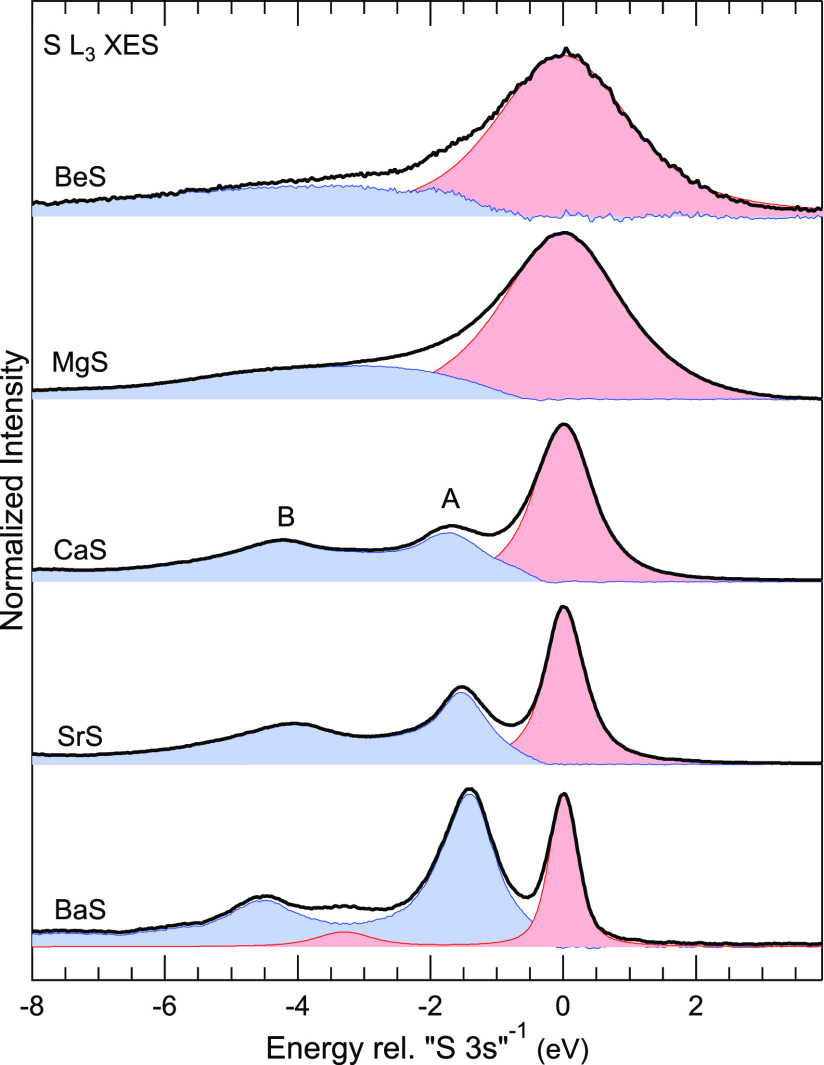
“S 3s” emission with satellites in the S L_3_ XES spectra of BeS, MgS, CaS, SrS, and BaS. The emission with a “S 3s”^–1^ final state is shown in red, and that of the satellites in blue. Energies are shown relative to the “S 3s”^–1^ final state. For CaS, SrS, and BaS, two separate satellite peaks are visible, labeled A and B.

For a more detailed discussion of the satellites, the spectra are shown in Figure 4 with separated contributions of the “S 3s”^–1^ final state (red) and the satellites (blue). To do this, the emission with “S 3s”^–1^ final state was fitted (in a range between 0.5 eV below to 4 eV above the peak maximum) with a sum of two Voigt lines at the same energy but with different widths and then subtracted from the overall spectrum to give the satellite contribution. For BaS, the contribution from the Ba 5p-derived band was accounted for with a separate symmetric peak at −3.3 eV, with an intensity ratio to the main line derived from the DFT-based spectra calculations. For BeS and MgS, the satellites appear as a broad structure, while the peaks are much narrower for CaS, SrS, and BaS, where we can distinguish at least two different satellite features (labeled A and B). We assign the satellites to “semi-Auger” (or “two-electron shakeup”) decays involving configuration interaction, as they were identified in the L_2,3_ emission of Cl to Cr.^14−19^ The final state for these satellites has a filled “S 3s” band, a double vacancy in the UVB, and one excited electron in the conduction band. Describing this in a simplified one-electron picture, the “S 3s” → S 2p_3/2_ transition is accompanied by an Auger transition, in which a UVB electron fills the “S 3s” hole and another UVB electron is promoted to the conduction band or above the ionization threshold.^17,19^

**Figure 5:**
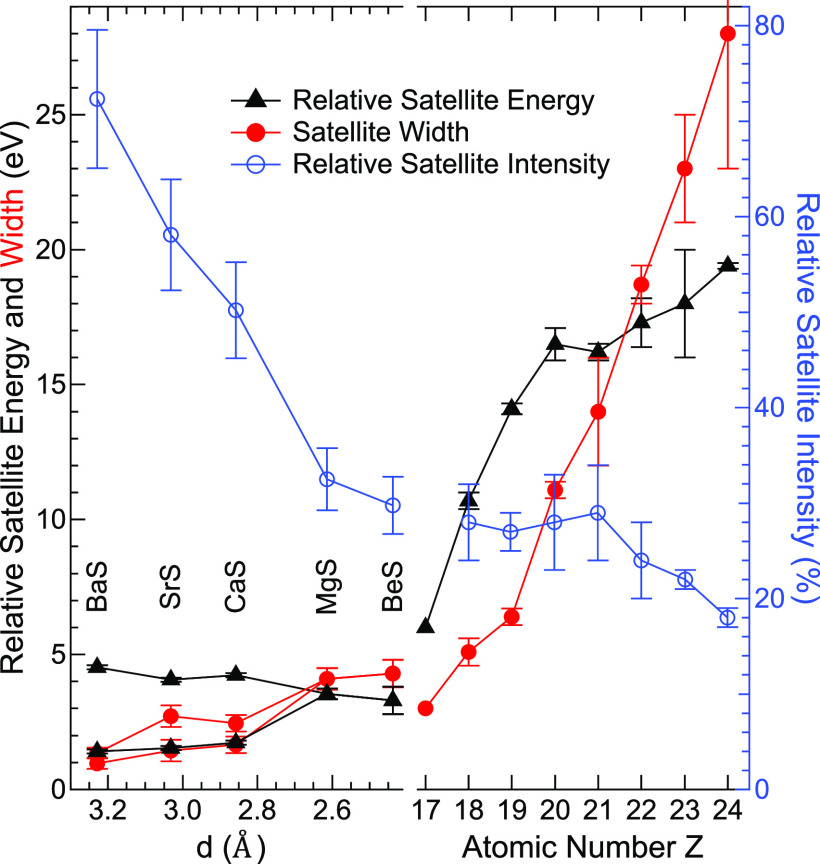
Relative satellite energies, widths, and intensities of the S L_2,3_ emission of the investigated alkaline earth metal sulfides (left, as a function of the lattice constant), in comparison with literature data^19^ of the satellite parameters for the elements Cl to Cr (right, *Z* = 17–24).

Velasquez and Schnatterly analyzed these satellites in the L_2,3_ emission as a function of atomic number *Z* of the emitting atom based on Hartree–Fock calculations of the configurations of the isolated atoms.^19^ In Figure 5, we compare their analysis (right half, as a function of atomic number *Z* from 17 to 24) with the values derived from our data (left half, *Z* = 16, as a function of lattice constant *d*). In agreement with their calculations, the energy loss of the satellite line observed by Velasquez and Schnatterly decreases from 19.4 eV for Cr (*Z* = 24) to approx. 6 eV for Cl (*Z* = 17). They argue that the energy loss approaches 0 below *Z* = 17 and the satellites vanish.^19^ Our S L_2,3_ emission data demonstrate that this is not the case. Furthermore, we find the satellite intensity, width, and energy to depend on the specific compound, as will be discussed in the following.

The satellite energy losses we observe in the S L_2,3_ emissions are smaller than the ones observed for higher *Z*, seamlessly extending the observed general *Z*-dependent behavior. However, we find the relative satellite position to differ significantly for the investigated compounds and can resolve at least two different satellite peaks for CaS, SrS, and BaS. The relative satellite energies of BeS and MgS lie somewhere between peaks A and B observed for CaS, SrS, and BaS, suggesting that two (or more) peaks are also present here but not resolved because of the widths of the features. BeS and MgS show a relative satellite intensity of approx. 30%, which extends the *Z*-dependent behavior observed by Velasquez and Schnatterly.^19^ However, the relative satellite intensity increases considerably for CaS, SrS, and BaS, reaching about 70% for BaS. As can be seen in Figure 4, this increase exclusively stems from an intensity increase of satellite A, while the intensity of satellite B does not change much. The model used by Velasquez and Schnatterly,^19^ based on the atomic properties of the emitting atom, can naturally not explain this behavior, as it does not take the differences in band structure of the investigated sulfides into account. Specifically, we find that the intensity increase of satellite A is accompanied by an energy shift closer to the “3s”^–1^ emission line, which might lead to a resonant increase of the probability of this satellite. We note that the satellites are equally present for non-resonant excitation (see Figure 1, upper half) and thus are not visibly influenced by the intermediate state of the X-ray emission process.

Finally, the widths of the satellite features for BeS and MgS roughly follow the *Z*-dependent trend. Again, we observe a strong dependency for the different alkaline earth metal sulfides—the widths decrease significantly with increasing lattice constant. In parallel, the line width of the “3s”^–1^ emission line decreases in a similar way. We attribute this to a change of the lifetimes of the respective valence hole states caused by the changes in the band structure. Specifically, and as discussed above, we observe a decrease in the band widths with increasing lattice constant. This in turn decreases the phase space for the (lifetime-determining) Auger decays and might thus lead to the observed decrease in line widths.

## Summary

Unusually strong satellite lines are found in the S L_2,3_ X-ray emission spectra of alkaline earth metal sulfides. The satellites are caused by “semi-Auger” decays with a double valence hole final state. Their energies, widths, and intensities strongly depend on the specific compound. Following the group of the periodic table from BeS to BaS, the most prominent satellite feature moves closer to the “S 3s” emission line, becomes narrower, and strongly increases in intensity. In the “S 3s” region of the spectrum of BaS, it accounts for 72% of the total intensity. Emission from the UVB region is also observed and can be well described by DFT-based spectra calculations. The relative intensity of this emission is very weak, owing to the predominantly p-type character of the involved bands and the dipole selection rules. Furthermore, the width of the band and its intensity in the spectra decreases when the bonding character of the compounds becomes more ionic (in the order BeS, MgS, CaS, SrS, and BaS). Finally, we note that only the spectra of BeS and MgS are quite similar, while the strong satellites and their compound dependency make S L_2,3_ XES a well-suited technique for the speciation of alkaline earth metal sulfides.

## Supporting Information Available

Description of the subtraction of higher order C K and O K signals from the spectra (PDF)
