# Supplementary material for: Satellite-Dominated Sulfur L2,3 X-ray Emission of Alkaline Earth Metal Sulfides
Source: ACS Omega. 2023 Jan 23;8(5):4921–7. doi: 10.1021/acsomega.2c07228 (PMC9909793; doi:10.1021/acsomega.2c07228)
Supplement: Supplementary file 1 — ao2c07228_si_001.pdf [file ao2c07228_si_001.pdf]

Supporting Information

Satellite-dominated sulfur $L_{2,3}$ X-ray emission of alkaline earth metal sulfides

Lothar Weinhardt,[1,2,3,] Dirk Hauschild,[1,2,3] Oliver Fuchs,[4] Ralph Steininger,[1] Nan Jiang,[3] Monika Blum,[3,5,6] Jonathan D. Denlinger,[5] Wanli Yang,[5] Eberhard Umbach,[4] and Clemens Heske[1,2,3]*

[1]Institute for Photon Science and Synchrotron Radiation (IPS), Karlsruhe Institute of Technology (KIT), Hermann-v.-Helmholtz-Platz 1, 76344 Eggenstein-Leopoldshafen, Germany

[2]Institute for Chemical Technology and Polymer Chemistry (ITCP), Karlsruhe Institute of Technology (KIT), Engesserstraße 18/20, 76128 Karlsruhe, Germany

[3]Department of Chemistry and Biochemistry, University of Nevada, Las Vegas (UNLV), 4505 Maryland Parkway, Las Vegas, NV 89154, USA

[4]Experimentelle Physik VII, Universität Würzburg, Am Hubland, 97074 Würzburg, Germany

[5]Advanced Light Source (ALS), Lawrence Berkeley National Laboratory, 1 Cyclotron Road, Berkeley, CA 94720, USA

[6]Chemical Sciences Division, Lawrence Berkeley National Laboratory, 1 Cyclotron Road, Berkeley, CA 94720, USA

Removal of C K and O K spectral contributions

As illustrated in Figure S1, the spectra of CaS, SrS, and BaS also contain contributions from C K and O K emission from the carbon tape substrate, excited by higher-order light of the beamline and collected in higher orders of the spectrometer. We find that these contributions are not present or negligible in the case of BeS and MgS. In the case of BeS, this can be attributed to a better

coverage of the carbon tape by the sample material and a lower efficiency of the employed (older) spectrometer for higher-order detection. In the case of MgS, a pellet was pressed, which fully avoids contributions from the substrate. For CaS, SrS, and BaS the C K (2nd order) and O K (3rd order) contributions can be well removed by subtracting a spectrum of each sample (red in Figure S1) excited just below the S L_3 absorption onset, thus only containing contributions from higher-order excitation. We note that this also removes a small fraction of the "true" sulfur $L_{2,3}$ emission, also excited by higher orders of the beamline (best seen at ~151 eV). Nevertheless, the subtraction routine works very well for emission energies below 155 eV, while artefacts are introduced in the region of the elastic lines. Thus, we only apply the subtraction for energies below 155 eV.

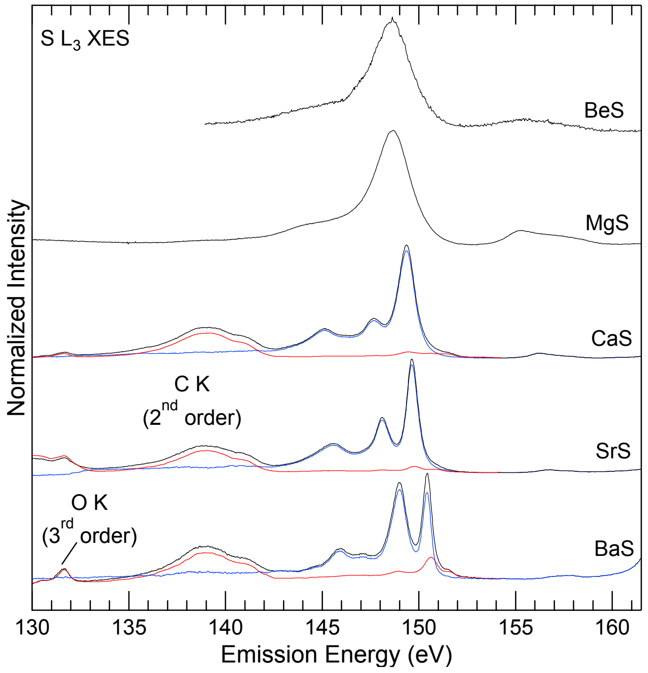

Figure 1: Subtraction of spectral contributions from C K and O K emission (excited by higher-order light from the beamline and detected in higher orders of the spectrometer grating) in the spectra of CaS, SrS, and BaS. For each sample, spectra excited below the L_3 absorption onset (red) were subtracted from the raw spectra (black), giving the corrected spectra (blue).
